# Redistribution and the health financing transition

**DOI:** 10.7189/jogh.11.16001

**Published:** 2021-11-20

**Authors:** Ajay Tandon, K Srinath Reddy

**Affiliations:** 1 World Bank Group, Washington, District of Columbia, USA; 2 Public Health Foundation of India, New Delhi, India

## Abstract

Public financing is necessary for realizing universal health coverage (UHC), a policy commitment that emphasizes that everyone should have access to health services they need, of sufficient quality to be effective, and that the use of these services does not expose individuals to financial hardship. As countries undergo their health financing transitions, moving away from external and out-of-pocket (OOP) financing toward domestically-sourced public financing, finding ways to increase public financing in an efficient, equitable, and sustainable manner is front and center in the policy dialogue around UHC. This paper focuses on one aspect of the health financing transition that has generally received less attention: that UHC is also intrinsically about a policy direction that emphasizes at its core redistribution of resources from the well-off to the poor. Differences in the level and organization of public financing for health for a given level of national income also reflect differences in social and political preferences for redistribution and equity across countries. Hence, navigation of a country’s health financing transition in ways that accelerates progress towards UHC also implies that public resources are targeted and expended in ways to improve effective service coverage and reduce OOP spending *specifically for the poor*. To leverage a country’s health financing transition for UHC, mechanisms should be introduced for: (i) ensuring that benefit entitlements are explicit and intertemporally commensurate with levels of public financing; (ii) fragmentation in pooling mechanisms is reduced to facilitate cross-subsidization without jeopardizing equity; (iii) levels of OOP and complementary sources of financing are nudged towards the well-off until core levels of public financing are adequate to provide similar levels of coverage for all; and (v) that purchasing of services is done in ways that helps reduce geographic- and income-related inequities in access and supply of quality health services. This implies careful policy choices need to be made, ones that require looking beyond the simplistic dichotomy between OOP and public sources of financing for UHC at the aggregate level to more nuanced and disaggregated assessments of the organization, use, and net fiscal incidence of financing and expenditures.

Universal health coverage (UHC)–a policy commitment that is part of the United Nation’s Sustainable Development Goals (SDGs) for 2030 – is about ensuring that all people can use the promotive, preventive, curative, rehabilitative, and palliative health services they need, of sufficient quality to be effective, while also ensuring the use of these services does not expose the individuals to financial hardship. The focus on both effective service coverage as well as financial risk protection under UHC implies that not only how much a country spends on health is important but also the way a health system is financed matters: for improving access for all in addition to ensuring protection from adverse financial and economic consequences resulting from illness which, for the poor and vulnerable, can be burdensome even at relatively low levels of direct out-of-pocket (OOP) spending at the time and place of seeking health care. Hallmarks of ‘high-performing’ health financing systems for UHC can be characterized as those where financing levels are adequate, prepaid funds are pooled in a sufficient way to spread the financial risks of ill-health, and spending is efficient and equitable to assure desired levels of effective service coverage and financial risk protection for all people, both with resilience and sustainability [1].

In its conceptualization, equity is intrinsic to the very notion of UHC: *everyone* should have access to services they need, and that access should be based on need and not on one’s ability to pay for health services. In addition, given that UHC is arguably more of a direction rather than a destination, equity concerns have led many to emphasize the notion of ‘progressive universalism’, that the poor and vulnerable ought to gain at least as much (if not more) while countries are on the path towards UHC [2]. Access, effectiveness, affordability, and equity are the hallmarks of a well-designed system headed towards UHC. While the first three attributes are relatively easy to define and measure, equity raises ethical questions in terms of the way the concept is applied with respect to the population served, services rendered, and costs covered, especially in the context of progressive universalization. Should efforts towards UHC treat all members of the population as eligible beneficiaries or assign greater priority to groups who are socially disadvantaged, have worse health indicators, and are financially more vulnerable? The concepts of ‘horizontal equity’ and ‘vertical equity’ become relevant while addressing this dilemma. Horizontal equity would enjoin us to treat all members of the population equally in terms of their entitlements, while vertical equity would aim to reduce the gaps in health status between population groups by giving greater attention to the groups that presently have a worse health profile and are economically disadvantaged [3,4].

This paper discusses UHC-related financing and redistribution challenges, providing country-specific examples where needed, in the context of economic growth and the change in status of countries from low-income to middle- and upper-middle income status, a change that is often accompanied also by a ‘health financing transition’, that is typically characterized by an increase in total health spending and a move away from external and OOP financing toward domestically-sourced public financing [5]. We argue that to leverage a country’s health financing transition for UHC, mechanisms should be introduced for: (i) ensuring that benefit entitlements are explicit and intertemporally commensurate with levels of public financing; (ii) fragmentation in pooling mechanisms is reduced to facilitate cross-subsidization without jeopardizing equity; (iii) levels of OOP and complementary sources of financing are nudged towards the rich until core levels of public financing are adequate to provide similar levels of coverage for all; and (v) that purchasing of services is done in ways that helps reduce geographic- and income-related inequities in access and supply of quality health services. This implies careful policy choices need to be made, ones that require looking beyond the simplistic dichotomy between OOP and public sources of financing for UHC at the aggregate level to more nuanced and disaggregated assessments of the organization, use, and net fiscal incidence of financing and expenditures.

The remainder of the paper is organized as follows. The Health Financing for UHC Section provides some background and context on health financing for UHC. Navigating The ‘Health Financing Transition’ For Accelerating UHC Section summarizes the health financing transition, including associated implications for redistribution of resources in the context of UHC. Country Experiences with Redistribution Section highlights some country experiences from a redistribution perspective. Paths Towards UHC summarizes with some key messages.

## HEALTH FINANCING FOR UHC

Health financing is both intrinsic to and instrumental for UHC. UHC is not only about increasing the number of people having access to health services, although this is clearly one important dimension, but also about ensuring that quality services are available and about financial protection accorded by health financing systems. Financial protection – ie, the extent to which citizens are protected from the risk of catastrophic health-related expenditure – is now widely recognized as one key objective of any health financing system [6,7]. From a revenue-generation perspective, country experiences have shown that compulsory prepayment modalities – especially general government revenue sources of financing–are necessary to ensure the progressive realization of UHC [8]. Financial protection requires risk pooling such that large, unpredictable health-related financial shocks that typically affect a small percentage of the pooled population in any given time period are substituted for by smaller, predictable prepayments (including taxes) of varying degrees incurred by all individuals in the pool.

Under OOP financing not only is there is no pooling of risks, it is also both an inefficient and inequitable means of financing health systems and antithetical to the very notion of UHC. OOP payments connect utilization of health services to an individual’s or household’s ability to pay; deter and delay utilization (especially for the poor), exacerbating or sustaining inequalities; and expose individuals or households to the risk of impoverishment resulting from high levels of health expenditures when they do utilize health services (constraining spending on other necessary expenditures). Given the general unpredictability and undesirability of health shocks and expenditures, OOP spending should generally only be used as a means for managing over-utilization and reducing waste and not as a core mechanism for generating revenues for health. OOP financing if often co-associated with a fee-for-service modality for provider payment which inherently incentivizing unnecessary utilization. High levels of OOP payments also reduce the potential to for using monopsony power to contain costs and improve efficiency. In addition, by their very nature, OOP payments constrain the overall redistributive capacity of health financing systems.

Prepayments under general government revenue financing take the form of direct and indirect taxes – on income, consumption, property, imports, etc. – that are pooled by governments and allocated across all sectors, including health. In some countries, prepayment and pooling of funds is also implemented via social health insurance (SHI) systems that are based on dedicated, mandatory payroll taxes with funds typically administered by parastatal organizations; even in countries implementing SHI programs, general government revenue co-financing has been critical for supply-side co-financing of public facilities and for subsidizing coverage for the poor and those in the informal sector [9]. Community-based health insurance and voluntary health insurance are other modalities of prepayment and risk pooling but these are generally not core for financing UHC because of their small scale and voluntary nature.

Given the importance of public financing more generally, and general government revenue financing more specifically, there is another aspect of UHC that receives less attention: that UHC is also intrinsically about a policy direction that emphasizes at its core redistribution of resources from the well-off to the poor. In addition to compulsion, subsidization for those not able to prepay is key for UHC [10]. Whereas compulsory prepayment combined with pooling of risk can enable cross-subsidization from the healthy to the sick, the design of prepayment and pooling mechanisms including subsidization of prepayments also allows for redistribution of pooled resources from the well-off to the poor, a necessary choice for many countries to ensure realization of UHC.

**Figure 1:**
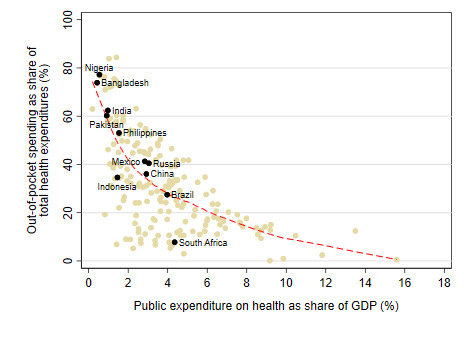
There is an inverse correlation between public and out-of-pocket (OOP) spending for health across countries [11].

Globally, there is a strong inverse relationship between the OOP and public financing for health (**Figure 1**; countries with population greater than 100 million highlighted). As can be seen, OOP spending remains the largest source of financing in several large developing countries including Nigeria, Bangladesh, Pakistan, India, and the Philippines. Countries that are closest to attaining UHC have OOP spending levels that are usually less than 15%-20% of total health spending (a threshold benchmark recommended by the World Health Organization (WHO) [12]; as, eg, is the case in high-income Organisation for Economic Co-operation and Development (OECD) countries) or where OOP spending–despite being higher than the 15%-20% threshold–is largely incident on well-off segments of the population and therefore is no longer a significant risk factor for impoverishment (eg, in Malaysia and Sri Lanka).

The discussion above implies that careful policy choices need to be made with regard to health financing for UHC, ones that require looking beyond the simplistic dichotomy between public vs OOP sources of financing for health at the aggregate national level to more nuanced and disaggregated assessments of the organization, use, and net fiscal incidence of financing. For example, even where public financing is large, the way it is organized and expended matters. If public financing is captured by the well-off, progress towards UHC will suffer; on the flip side, countries may have low levels of OOP financing simply because this is reflecting foregone care by the poor. Smaller, fragmentated pools of financing are generally less effective than larger diverse pools; the former can contribute to inequities by preventing redistribution of resources. Similarly, even though levels of OOP financing may appear large at the aggregate national level in some countries, they may be less problematic if they are incident on the rich and are the result of mechanisms to target scarce public financing resources towards the poor. How public resources are pooled and expended – including what services are financed, how explicitly these are specified, and how providers are paid – are important from both efficiency and equity perspectives as country’s navigate their health financing transitions. Although the arguments we make look at equity from the perspective of those who are income poor, these are equally relevant for other vulnerable population sub-groups and along other dimensions such as gender.

One important caveat: in assessing redistribution of public resources, it is conceptually more correct to focus on the *net* fiscal incidence of overall public financing – both in terms of how revenues are raised and how expenditure is expended–and not just on incidence of public financing specifically for health. It is conceivable is some situations that public resources for health are raised in a regressive manner but in the end – when benefits are considered – the net incidence is progressive if the funds are targeted appropriately. Similarly, there may be situations whereby public financing specifically for health is not incident on the poor but that the poor are compensated via other forms of redistribution, eg, via income transfers such that the net fiscal incidence is progressive even if the incidence for health is not. Similarly, overall public revenue generation may be progressive; however, if the benefit-incidence of public spending is pro-rich, then looking just at ‘one side of the coin’ can give the wrong impression on the equity dimension of public resources. For example, raising tobacco taxes may impose a greater financial burden on the poor who generally constitute the largest fraction of tobacco consumers. However, they will also stand to benefit the most in terms of health gains by the raised price driving down consumption. Moreover, the extra revenue generated by an increased tobacco tax can be expended to enhance the public funding for UHC, either through a higher allocation from an expanded general revenue pool or through specifically directed allocation. For example, Philippines has raised tobacco taxes and dedicated 85% of the increased revenue to health and 80% of that to UHC [13].

## NAVIGATING THE ‘HEALTH FINANCING TRANSITION’ FOR ACCELERATING UHC

Economic growth and development are usually accompanied by many different and significant transitions related to health. For instance, countries tend to undergo a *demographic* transition as national incomes rise: a decline from high mortality and fertility rates to relatively low mortality and fertility rates that result in ageing of the population. Related in part to the demographic transition, countries also tend to undergo an *epidemiological* transition: a change in the pattern of the overall disease burden from one that is dominated by maternal and child health problems as well as communicable diseases towards one were chronic non-communicable diseases (NCDs) are prominent. Furthermore, countries also undergo a *nutrition* transition which typically refers to a move away from problems related to undernutrition towards those that are attributable to being overweight and obese.

In parallel with the demographic, epidemiological, and nutrition-related transitions faced by countries as they grow and develop, there is also what some have called a health financing transition: the tendency for the levels of health expenditure to increase, accompanied by an increase in the domestic publicly-financed share of health spending; the flip side being a decline in the external- and OOP-financed share of health spending as national incomes rise [5]. These empirical trends are driven by a range of factors: institutional developments, medical technological advancements, ageing, changing population preferences, etc. Some influence the overall quantum of health spending while others impact the way in which health systems are financed.

**Figure 2:**
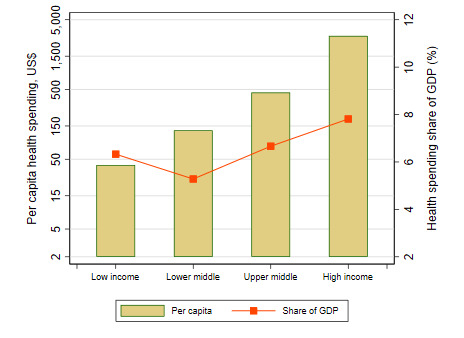
Total health spending generally increases with national income, both in levels and as share of GDP [11].

**Figure 3:**
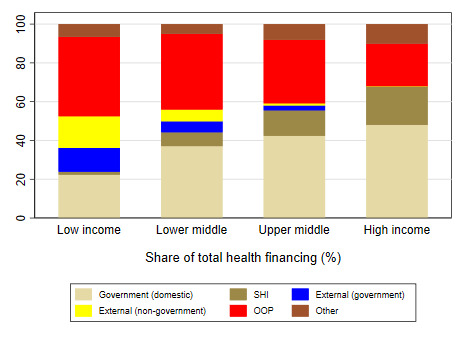
Sources of financing for health also change with national income [11].

The health financing transition is evident in cross-country data: low-income countries (LICs) spent an average of US$37 per capita in 2017, or 6.2% of GDP. Richer countries spent far more: lower middle income (LMI) countries spent US$128 (5.3% of GDP), upper middle income (UMI) averaged US$449 (6.3% of GDP), non-OECD high-income countries (HICs) spent US$1293 (5.4% of GDP), and OECD HICs spent US$3733 (9.6% of GDP) (**Figure 2**). Also, the sources of health financing change with national income: HICs financed most of health spending vis public sources of finance – a mixture of domestic general government revenues and SHI contributions – whereas lower income countries were predominately financed from OOP sources (**Figure 3**).

The health financing transition describes an empirical trend that reflects what tends to happen on average as countries move up the income ladder. There are important differences, though, across countries and many factors can shape the timing and magnitude of the transition and the extent to which it poses a policy challenge, especially in LMICs. For example, in most Pacific countries, OOP financing has traditionally been low, and so the major challenges faced by those countries is keeping OOP low during the transition from external to domestic public sources of financing. In other developing countries, the challenge is about replacing both external and OOP sources with domestically-sourced public financing in order sustain progress towards UHC. In countries such as Myanmar, political events have led to the paradoxical situation whereby external financing for health has increased despite high levels of economic growth and despite the country’s recent transition from low- to lower-middle income status.

**Figure 4:**
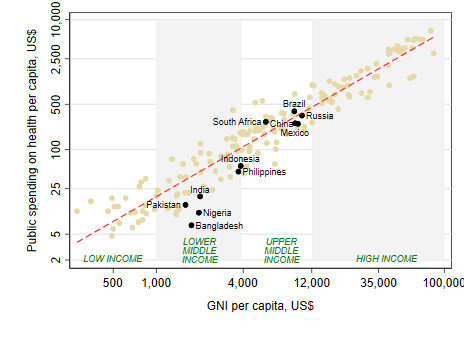
Public spending on health closely follows national income [11,14].

**Figure 5:**
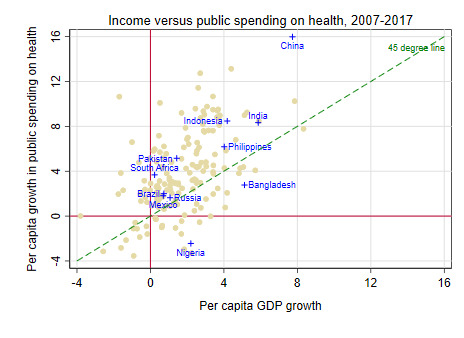
Growth in public spending on health vs economic growth [11,14].

Countries often struggle in making progress in their health financing transition due to lack of adequate public financing for health which, in turn and in large part, is driven by three key factors: economic growth, revenue-raising capacity of governments, and prioritization of health within government budgets. Levels of public financing for health are highly correlated with levels of national income across countries (**Figure 4**). On average, public financing tends to increase in line with increases in national income. And countries that have experienced high levels of economic growth generally see levels of public financing for health increasing at faster rates. India is a case in point: although levels of public financing for health remain far below those expected for its income level, relatively high economic growth rates have resulted in almost a tripling in real public financing for health in per capita terms since 2000 [15]. On the flip side, developing countries on a lower-growth trajectory – eg, Nigeria, Mexico, Russia, and Brazil – have faced a more constrained environment for increasing public financing for health (**Figure 5**).

**Figure 6:**
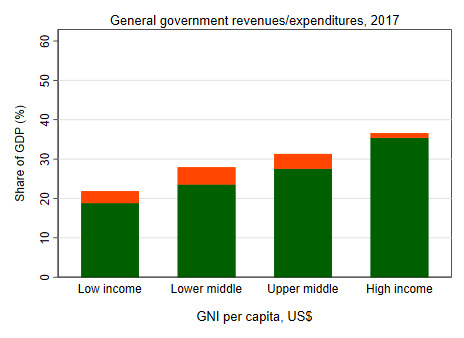
Government revenues and expenditures rise with income [16].

**Figure 7:**
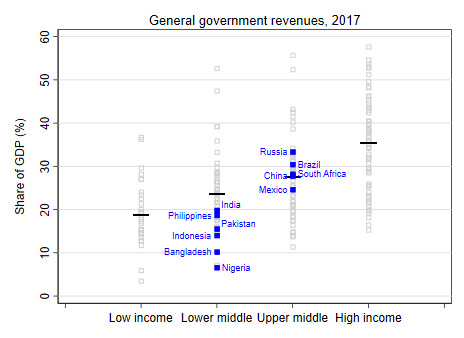
Government revenues as share of GDP [16].

A second factor that impacts the level of public financing for health (and, in fact, all other sectors) is the revenue-generating capacity of governments. Lower income countries tend to have lower levels of government revenue shares of GDP, in part due to higher levels of poverty, informality, and lower institutional and other capacities to collect direct taxes. Whereas in HICs, general government revenues were more than 35% of GDP, this number was less than 20% in LICs (**Figure 6**). Even within country income groups, there are large variations: the government revenue share of GDP is especially low in countries such as Nigeria and Bangladesh, constraining their ability to publicly finance spending across all sectors, including health (**Figure 7**).

**Figure 8:**
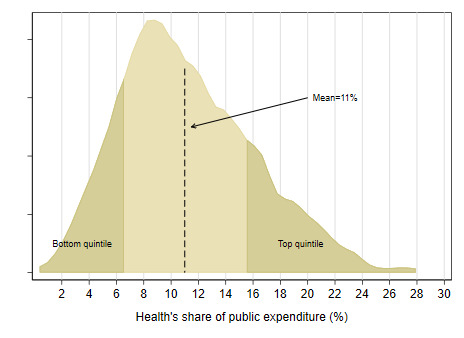
Distribution of health’s share of government expenditure [11].

Furthermore, countries vary widely with respect to the share of health in public spending, a metric that can serve as a crude proxy for the extent to which health is prioritized by governments. Globally, the average share of health in public expenditures stands at about 11% (**Figure 8**). However, there are large and notable variations across countries: health accounts for less than 3% of public expenditures in Venezuela, Iraq, and Equatorial Guinea to almost 30% in Costa Rica. Countries where health’s share is below 6% (such as Bangladesh, India, Myanmar, Cameroon, Egypt, Lao PDR, Nigeria, Afghanistan, Egypt, Haiti, Cambodia, Cote d’Ivoire, and Uganda) are in the bottom quintile whereas those where health’s share is greater than 15% (such as Peru, Tanzania, Sierra Leone, Malawi, Rwanda, Madagascar, Guatemala, Costa Rica, and Colombia) are among the top quintile globally. In comparing prioritization across countries, it is important to note that the relationship between health’s share of public expenditure and public financing for health as a share of GDP is not monotonic since the size of public expenditures are different across countries. A country such as Cuba has a lower health share of public expenditures relative to Iran but a higher share of GDP because its size of public expenditures is higher. Indonesia, on the other hand, has roughly the same health share of public expenditures as Bhutan but is lower as share of GDP because the size of public expenditure in Indonesia is lower than that of Bhutan’s.

Despite large country-specific variations, health’s share of public expenditure does tend to vary systematically by a country’s income classification and region. As might be expected, affluent countries are more likely to prioritize health and, over time, there appears to have been a small secular increase in prioritization in recent years that has impacted countries across all income classifications. Relatively high levels of deficits, debt, and military expenditure can compound the problem of prioritization of public financing across countries, including for health [17].

**Figure 9:**
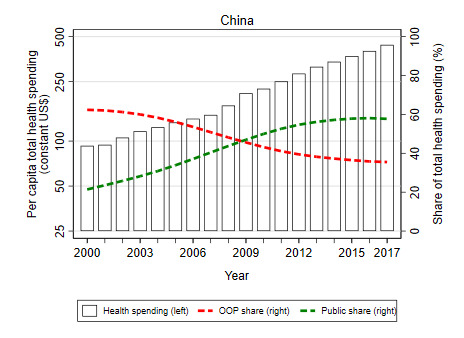
China’s progress towards its health financing transition [11].

**Figure 10:**
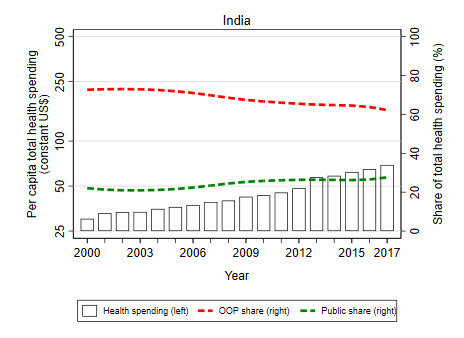
India’s progress towards its health financing transition. Source: WHO, 2020 [11].

The example of China and India underscore potential differences in health financing trajectories of countries over time. For example, China appears to be well-advanced in its health financing transition with a rising share of public financing offsetting a declining share from OOP sources due to economic growth, rising general government revenues, and reprioritization of health; this is not the case for India where public financing for health has remained a relatively low share of overall health spending despite increasing significantly in levels in recent years, and OOP sources predominate (**Figure 9**). India’s lack of progress on its health financing transition is largely due to low priority given to health in the national budget (**Figure 10**). If and how countries undergo their health financing transition – especially in terms of the willingness and ability of countries to increase public financing for health – will largely determine the rate of progress toward UHC for decades to come. Hence, improving an understanding of some of the factors that can accelerate a country’s health financing transition – including the willingness and ability of countries to redistribute public resources for health–is important from a policy perspective.

The concept of UHC encompasses a wide range of services: including those that are promotive, preventive, diagnostic, therapeutic, rehabilitative, and palliative. A focus under expansion of UHC for curative care while neglecting promotive and preventive services will be regressive because the poor have the highest risk of developing diseases if such services are deficient. This places the poor at a higher risk of suffering financial hardships. Some of the elements of disease prevention and health promotion fall outside the ambit of the conventional health sector such as water, sanitation, environment, food, and agricultural systems. Though the conceptualization of UHC does not explicitly cover such determinants of health, it is imperative that public financing is also available at adequate levels for health-enabling actions in other such domains. This broader approach may be regarded as ‘UHC+’. Combined with a progressive universalism that prioritizes the poor and vulnerable at every stage of its evolution, the multi-sectoral actions that protect, preserve and promote health enhance both the horizontal and vertical dimensions of equity.

**Figure 11:**
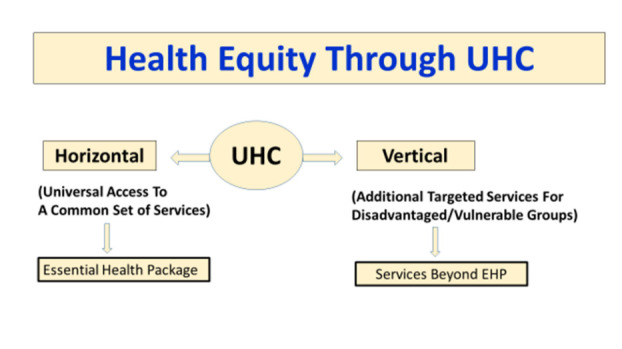
Health equity and universal health coverage (UHC).

Since public financing for UHC is limited in developing countries, likely to progressively increase over time as countries grow and develop, choices will need to be made as to how best to address equity at each stage. To be truly universal, population coverage has to be complete for at least some services to begin with. Comprehensive primary care services (including NCD and mental health related services), emergency services, maternal and child health services as well as some surgical services would qualify for early inclusion in a universally-guaranteed package. If countries dedicate a high level of public financing, this package can be larger even to begin with. The availability of a universally-guaranteed package, where services are available through prepaid pooled financing reflects a commitment to horizontal equity. An additional package of services and/or additional cost coverage may be provided simultaneously to poor and vulnerable population groups. This promotes vertical equity (**Figure 11**). These two dimensions of equity will need to be continually addressed as more public resources become available. As the ‘horizontal package’ expands in service and cost coverage at each incremental stage, so will the ‘vertical package’ of additional services and financial protection provided to the poor and vulnerable sections.

Similarly, since primary care is arguably a universal health need, where every individual requires some form of primary care over the course of their lifetime – often for a prolonged period in case of NCDs and mental health disorders – OOP payments for primary care can have markedly adverse effects on the poor as they deter and delay utilization of services before complications arise. A high level of public financing for primary care, arguably, signals a commitment to a UHC with a redistributive emphasis. Public funding for an efficient and universally accessible primary care system also reduces the need for advanced secondary and tertiary care which is expensive and often requires hospitalization. Not only will the poor be less vulnerable to the catastrophic hospitalization-related expenditure and overall societal savings can also help realize efficiencies in how scarce public funds are used.

As noted earlier, the process towards UHC is just as important as the outcome, and some health financing choices can make the poor worse-off in the short- to medium-term, so having an equitable pathway is just as important as the final objective of UHC. In recent debates around this issue, ten unacceptable trade-offs have been highlighted to help inform the design and implementation of health financing policies as summarized in **Table 1**. These include unacceptable trade-offs related to how health systems are financed, eg, it is not acceptable to raise UHC revenues via voluntary prepayment mechanisms or from OOP sources, as well as trade-offs related to how benefits are distributed, eg, designing benefits and allocating resources in ways that exacerbate inequities.

**Table 1:** Ten unacceptable trade-offs linked to health financing [1]

	It is unacceptable to:
Financing contributions to the system	1. Increase OOP payments with an exemption system or compensating mechanism for the poor
	2. Raise additional revenues for health in ways that make contributions to the overall public financing system less progressive
	3. Raise revenues for universally-guaranteed benefits through voluntary prepaid pooled financing arrangements that are based on health status, including pre-existing conditions and risk factors
Benefits from the system	4. Change allocations across prepaid pooled schemes that worsen inequalities
	5. Change allocations across different levels of administration that worsen inequalities
	6. Within prepaid pooled schemes, change allocations across diseases that worsen inequalities
	7. Introduce high-cost, low-benefit interventions to a universally-guaranteed package before close to full coverage for high-benefit, low-cost series has been achieved
	8. Increase the availability of quality personal health services in ways that exacerbate inequalities
	9. Increase the availability of quality public health services in ways that exacerbate inequalities
	10. Increase the availability of inputs in ways that exacerbate inequalities

OOP – out of pocket

## COUNTRY EXPERIENCES WITH REDISTRIBUTION

Most developing countries have at some point or another started from a basic system whereby the government owns, finances, and provides health care services: wherein public financing from general government revenues flows to health ministries from the ‘supply-side’ as in the traditional Beveridge-style models prevalent in the UK, Italy, Norway, and Scandinavian countries or the more state-controlled Semashko-style model in many ex-Soviet Union countries. At least theoretically, such systems allow for a redistribution of public financing for health as long as the net benefit-incidence for the poor or other vulnerable groups is positive, eg, when contributions in the form of general government revenues share collected from the poor and proportionally allocated to health are less than the incidence of public spending that goes into directly benefiting this group. From the revenue side, it remains up to the general tax collection system to ensure equitable contributions in public financing more generally. From the expenditure side, many countries introduced user fees for the non-poor to access public facilities and benefits – sometimes implemented by providing hoteling differences such as charge-back access to private rooms – as a way to preserve the redistributive intent of such financing mechanisms.

One problem that can and has occurred in many Beveridge- and Semashko-style systems of public financing and delivery of health is when the geographic distribution of facilities and services is such that access to them by the poor gets impeded. A classic example being a situation when higher-end and more complex secondary and tertiary hospital services provided by the public sector are skewed in the placement of facilities that provide such care towards urban areas that limits access to the rural poor. This can result in a pro-rich benefit incidence for hospital services that reverses the redistributive intent of public financing and contributes to income- and geographic inequalities in access and outcomes, even when offset by transportation and other benefits provided to the poor to offset the spatial maldistribution of facilities. A recent assessment in Indonesia found that the richest 20% of the population accounted for 25% of all inpatient discharges across public hospitals in the country, whereas the bottom 20% accounted for only 16%: a relatively pro-rich level of participation incidence [18].

A second problem with supply-side systems of public financing has been one of chronic under-financing which has, in turn, resulted in implicit rationing of services, poor quality of care, and prevalence of informal payments, the adverse impacts of which generally fall disproportionally on the poor. In some cases – as in the case of India, Pakistan, and Bangladesh – this has contributed not only to a large-scale privatization of financing in health care but also of service delivery, the latter often being of highly variable quality and contributing to fragmentation and exacerbating inequalities in outcomes [19].

Nevertheless, some countries such as Sri Lanka and Malaysia have managed to successfully implement pro-poor redistributive tax-financed and publicly-provided health care services, with the private sector catering to the well-off by providing more responsive care. The poor are able to access relatively good-quality health services in public facilities; the well-off self-select out of the public system to access OOP-financed care in private settings that requires less waiting time and provides better hoteling services. The key to success in such systems has been preserving supply-side readiness and quality of care in public facilities.

More recently, many countries have introduced or expanded ‘demand-side’ insurance-style public financing where money follows the patient and not the facility. This form of financing – a variation of the Bismarck-style SHI model that was introduced in Germany in the late 1800s based on mandatory earmarked payroll-based contributions – implements redistribution of public financing via how contributions are set and how benefits packages are designed. Variations of such SHI programs are being implemented in many developing countries such as Vietnam, Philippines, and Indonesia that collect mandatory wage-based contributions from the formal public and private sector, pool them with government-financed premiums paid on behalf of the poor, and use these prepaid pooled funds to purchase health service from both public and private providers.

It is notable that, on average, SHI contributions are a small share of revenues for the health sector in LICs and LMICs, primarily due to high rates of informality and poverty [20]. This context is hard to change without progresses in other development areas such as in increasing labor-market formality, governance, and institutional development. Global experience has shown that collecting contributions from the non-poor informal sector has been an almost-insurmountable challenge. Thailand struggled for many years to collect contributions from the non-poor informal sector, eventually abandoning this strategy in 2001 to provide non-contributory coverage to 75% of its population ever since. Indonesia’s SHI program has faced similar challenges of adverse selection in enrollment of the non-poor informal sector; as a result, the country has been slowly expanding tax-financed coverage to the near-poor in a bid to attain UHC.

Many countries including China and Turkey initially began with similar programs that only provided tax-financed inpatient care coverage for the poor and vulnerable; such arrangements became the basis for subsequent expansion of benefits to primary care and of population coverage to other sub-groups by merging them with contributions from formal sector premium payments. If designed correctly, such programs allow for redistribution that is implicit (under the tax-financed portion of revenues) and explicit (under the SHI contributions portion of revenues). Mergers with formal sector programs have the advantage of reducing fragmentation from a political point of view, such that the relatively well-off also have a direct vested interested interest in seeing the programs succeed. Other forms of demand-side expansion have focused solely on tax-financed implementation of benefits packages, without any contributions from SHI sources. Current reforms in Ukraine, for example, are being implemented to provide a prioritized benefits package of services to all based on general government revenue financing: similar in structure to the systems in place in Canada and Latvia, among other countries. On the flip side, some countries have introduced demand-side SHI systems only for the formal sector, leaving the rest of the population to seek care in the traditional line-item budget (under)financed public system or use voluntary insurance and OOP payments to seek care in the private sector, adding to the fragmentation of health financing and constraining the redistributive capacity of the system.

From a redistributive perspective, demand-side financing programs remains vulnerable to the same issues and challenges as those under traditional supply-side financing systems: if under-financed, such systems encourage informal payments and gaming of the system by providers. Where benefits packages are not explicit and not commensurate with the levels of public financing, implicit rationing remains a big problem. Where geographic inequalities in access to facilities and benefits packages remain, such programs risk ‘reverse redistribution’: with the poor subsidizing access by the rich as has recently emerged in Indonesia’s SHI system [18]. Where there are large levels of informality in the labor force, making it difficult to collect contributions and implement contribution mandates, there are issues of adverse selection and disproportionate access to benefits by the non-poor informal sector raising concerns with regard to the benefit-incidence of public funds. Some of these problems can be offset by implementing tweaks in the benefits package – eg, as in Kyrgyz Republic and Armenia – where the rich have to pay large amounts of co-payments to access the same package of services that the poor can access for free, but this has contributed to high levels of OOP payments in these systems that in of itself has constrained the redistributive capacity of a health financing system.

Regardless of the demand-side vs supply-side plumbing of public financing, it is important to have an explicit benefits package to enhance transparency and accountability, especially from a redistributive perspective. For example, Kyrgyz Republic provides a publicly-financed State Guaranteed Benefits Package (SGBP) that is a non-contributory universal entitlement for a basic explicit package of primary health care services. OOP payments for inpatient and specialized care – which are not covered under SGBP–are exempt for vulnerable groups and are paid for by others via participation in a contributory Mandatory Health Insurance (MHI) scheme. Indonesia, on the other hand, has an open-ended non-explicit benefits package that is supposed to cover all services that are deemed medically necessary under its national SHI program; lack of an explicit package and low levels of public financing have led to a situation whereby the full range of benefits are only available under JKN in large urban centers, in effect leading to implicit rationing of services in rural and poorer areas of the country.

## CONCLUSIONS

There is no one monolithic health financing model that works across all countries, and countries have varied in terms of how they have implemented redistribution of public financing from an equity perspective. Some countries such as Sri Lanka and Malaysia have excellent tax-financed and publicly-provided health care services, with the private sector catering to the well-off by providing more responsive care; other countries such as Thailand have implemented tax-financed demand-side coverage by separating purchasing and provision in order to integrate public and private provision of services. Countries such as Indonesia, Philippines, and Vietnam use a mix of contributory coverage for the formal sector and tax-financed non-contributory coverage for the poor that is pooled to purchases health care from both public and private providers. Regardless of the design of a health financing system, country experiences point to some basic principles that are evident that can help offset some of the challenges related to implementing the redistribution objective of public financing for health within the context of navigating a country’s health financing transition for accelerating progress towards UHC. Some of these are reiterated and summarized below.

*Navigating a Country’s Health Financing Transition for UHC Implies An Explicit Focus on Redistribution*: In order to make progress on UHC, public financing for health will need to increase and be expended in ways that focus on improving effective coverage and financial protection, especially among the bottom 40% of the population. In addition to increasing the level of financing, improvements in efficiency and equity of public expenditures for health will also needed. This implies moving allocations away from secondary and tertiary hospitals towards frontline and primary health care services, especially in rural and poorer parts of the country. Countries cannot spend their way to UHC, and careful targeting of scare public resources will be necessary to optimize effectiveness, especially scaling up of both traditional interventions targeting preventable maternal and child health conditions as well those geared towards addressing the rising the burden of NCDs.

*Redistribution Objectives Can Be Attained from Both a Revenue and Expenditure Perspective*: At its core, UHC is also about redistributing resources from the well-off to the poor. Redistribution objectives can be attained by adjusting both the incidence of revenues as well as expenditures to ensure that health financing systems are reducing inequalities by emphasizing vertical equity considerations over horizontal equity objectives where necessary.

*General Government Revenues Are Key for Financing UHC*: Global experience underscores the need to build a strategy for UHC with general government revenue financing for health at its core, one that recognizes that contributions from other sources of revenues such as earmarking, SHI, and cost-sharing arrangements are likely to be symbolic and marginal at best. Collecting contributions from the non-poor informal sector has been an almost-insurmountable challenge, one that almost all countries have found difficult. Thailand struggled for many years to collect contributions from the non-poor informal sector, eventually abandoning this strategy in 2001 to provide non-contributory coverage to 75% of its population ever since. Indonesia’s SHI program has faced similar challenges of adverse selection in enrollment of the non-poor informal sector; as a result, the country has been slowly expanding tax-financed coverage to the near-poor.

*Public and OOP Financing for Health are Often Two Sides of the Same Coin*: High-levels of OOP are often the flip side of low levels of public spending on health. High levels of OOP and low public spending on health are both drivers of inefficiencies and inequities. However, both the levels and incidence of financing matters: large levels of public financing that are captured by the rich, and low levels of OOP financing that reflect foregone care by the poor are just as undesirable as low levels of public financing and high levels of OOP financing. This implies careful policy choices need to be made, ones that require looking beyond the simplistic dichotomy between OOP and public sources of financing for UHC at the aggregate level to more nuanced and disaggregated assessments of the organization, use, and net incidence of financing and expenditures.

*How a Health System is Financed is Strongly Linked to the Economy*: How a health system is financed matters for economic outcomes, including from the perspective of reducing impoverishment, increasing productivity, and stimulating economic growth. However, a country’s overall macro-fiscal context also matters for its ability to publicly finance health and other programs. Where public financing for health is constrained, it should be targeted towards the poor via vertical equity objectives, letting OOP and other complementary sources of financing be incident on the well-off. Having a clear publicly-financed benefits package that is commensurate and intertemporally adjusted over time with public financing would remove implicit rationing of services and enhance accountability.
